# Supplementary figures and images for: Library-based analysis reveals segment and length dependent characteristics of defective influenza genomes
Source: PLoS Pathog. 2021 Dec 9;17(12):e1010125. doi: 10.1371/journal.ppat.1010125 (PMC8691639; doi:10.1371/journal.ppat.1010125)

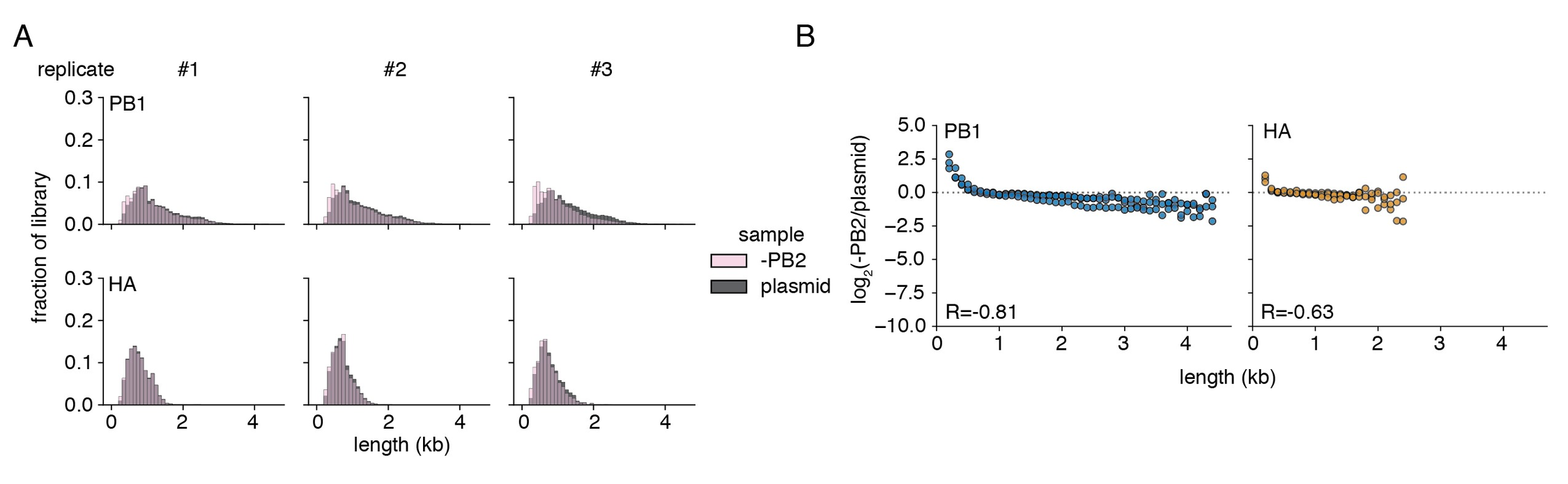

Supplement: S1 Fig — Biases present from polymerase I transcription alone. (A) Size distributions of original plasmid libraries and matched length distribution after transfection in the absence of the full viral genome replication machinery (-PB2). (B) Polymerase I transcription alone leads to a slight length bias in library composition. To analyze enrichment, or depletion, the fraction of variants falling within each 100nt bin was compared between replication-incompetent selection and the original plasmid library. Points above the dotted line represent lengths which were overrepresented in our polI transcribed library relative to a plasmid control. Points were only shown if represented in all three libraries under both conditions. R-value shown is the Spearman correlation coefficient. n = 3. (TIF) [file ppat.1010125.s006.tif]

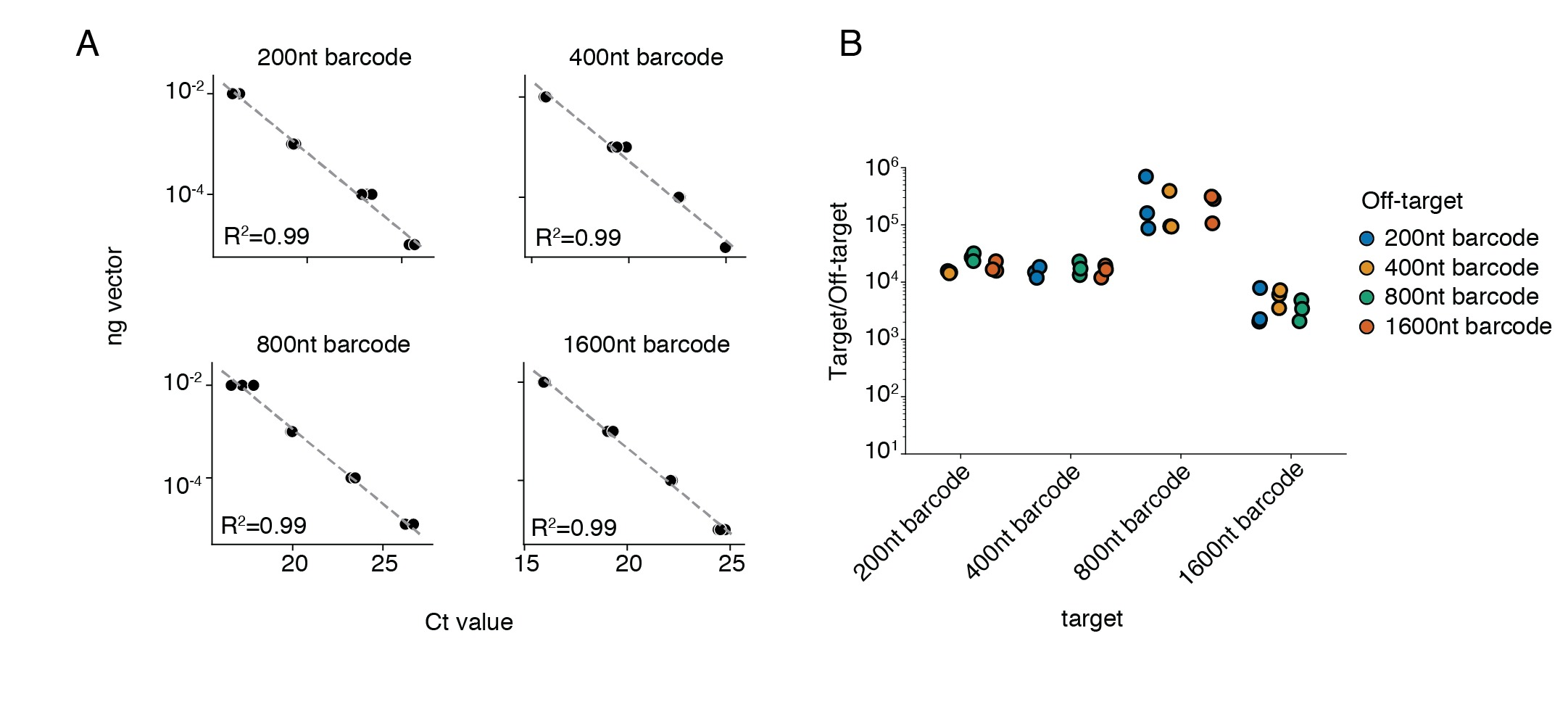

Supplement: S2 Fig — (A) All four qPCR reactions exhibit linearity when tested against a plasmid control. R2 from linear regression against Ct verus the log-transformed plasmid concentration. Linear regression line shown. (B) Each qPCR is highly-specific to its cognate target, and discriminatory against non-cognate targets. Target versus non-target specificity was calculated as the relative signal of each qPCR reaction on the indicated off-target control when compared to a target control at 0.01 ng per qPCR reaction. n = 3 for both panels. (TIF) [file ppat.1010125.s007.tif]

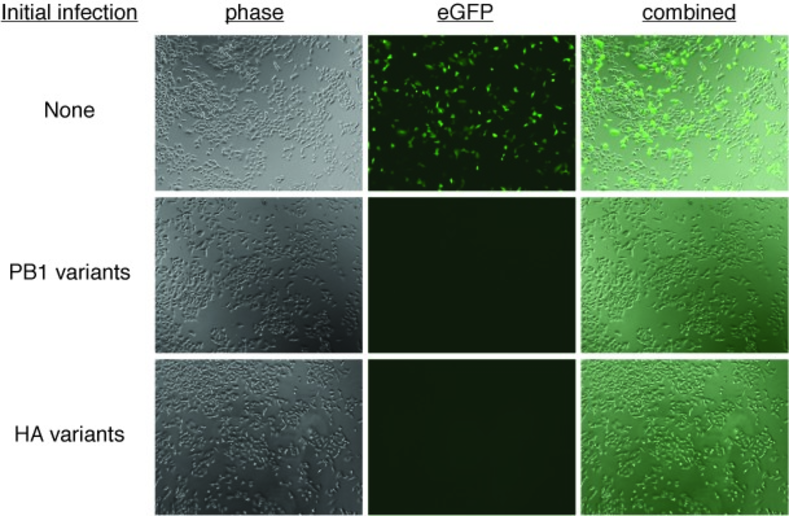

Supplement: S3 Fig — Infections were performed as in Fig 2D, save for, at 8 hours post infection, cells were infected with a pseudovirus wherein the coding sequence for HA has been replaced by eGFP, with packaging signals maintained. [64] Micrographs were taken 16 hours post infection with HA-flanked eGFP pseudovirus. A lack of fluorescent cells in our variant infections indicates that, at least largely, second rounds of replication are excluded under these high MOI conditions via superinfection exclusion. (TIF) [file ppat.1010125.s008.tif]

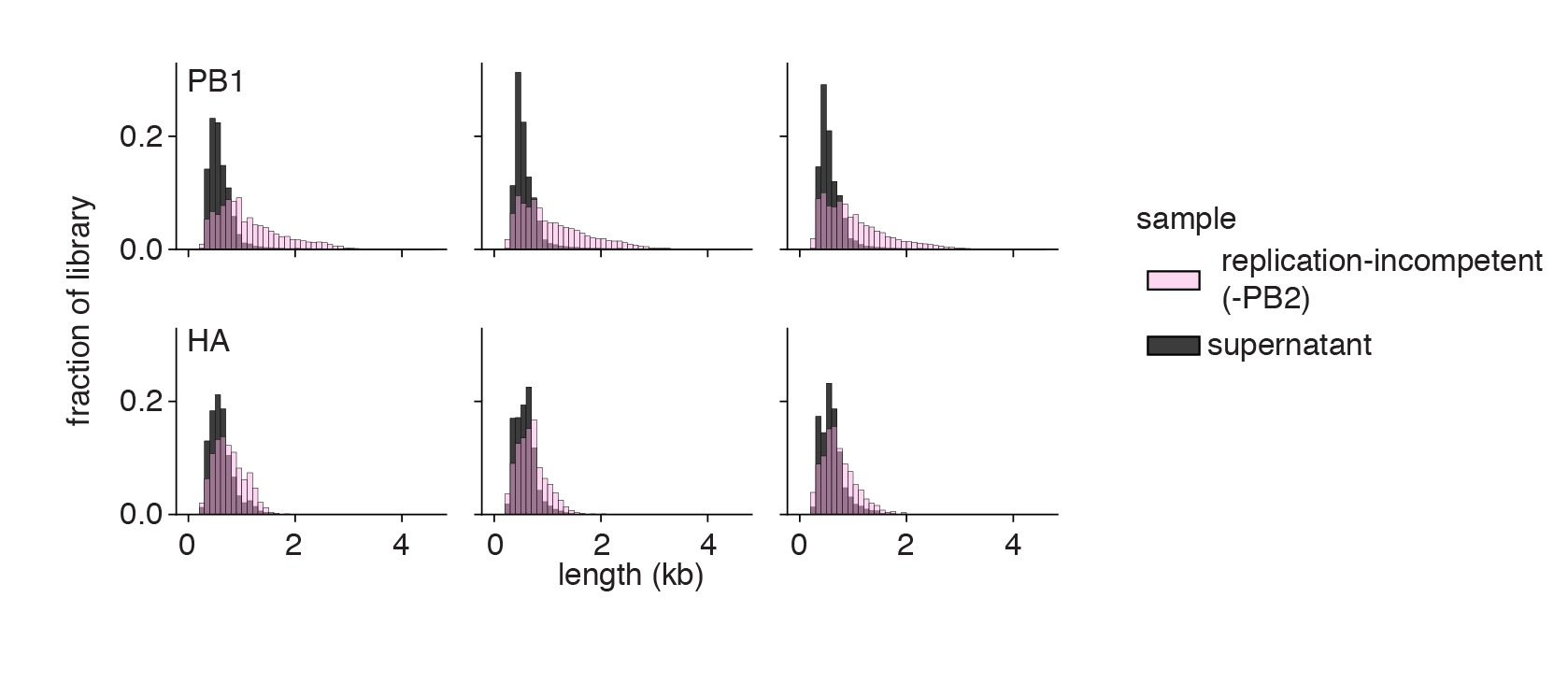

Supplement: S4 Fig — (TIF) [file ppat.1010125.s009.tif]

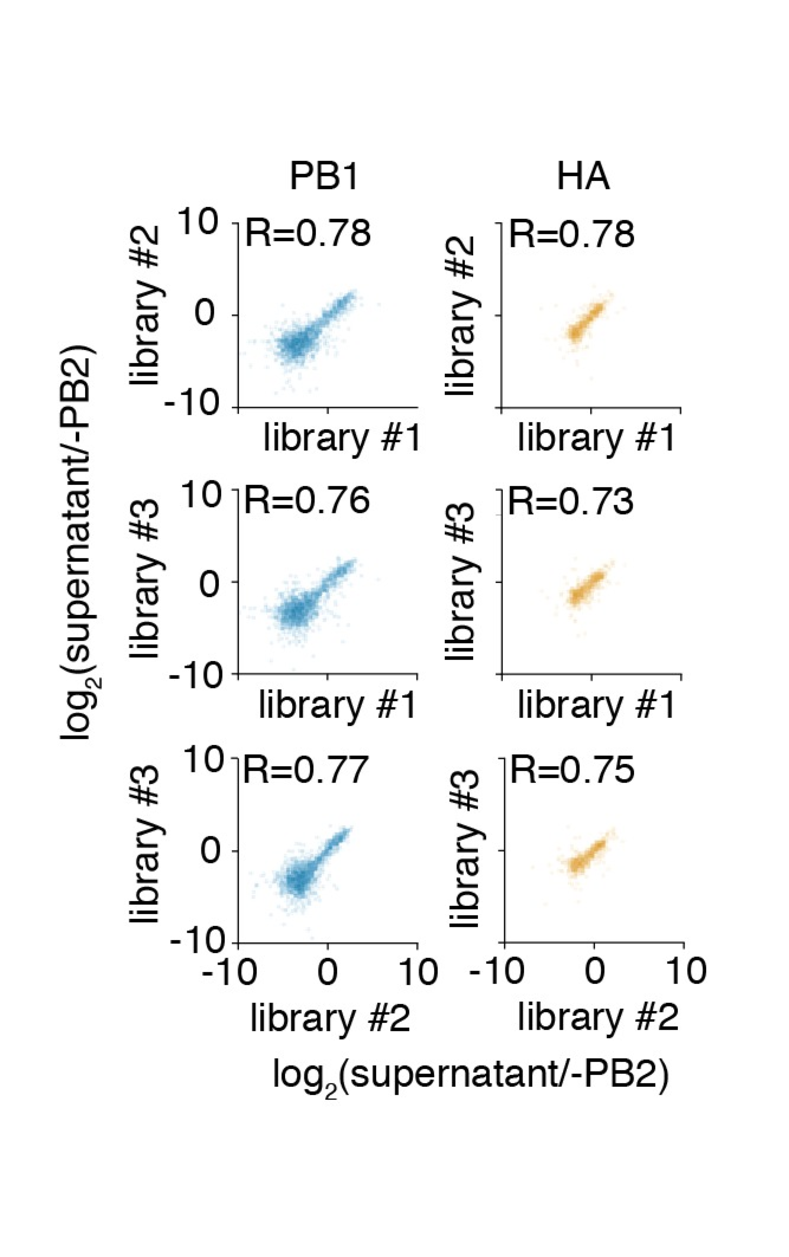

Supplement: S5 Fig — Inter-replicate enrichment values as calculated in Fig 3E. R is the Pearson correlation coefficient. (TIF) [file ppat.1010125.s010.tif]

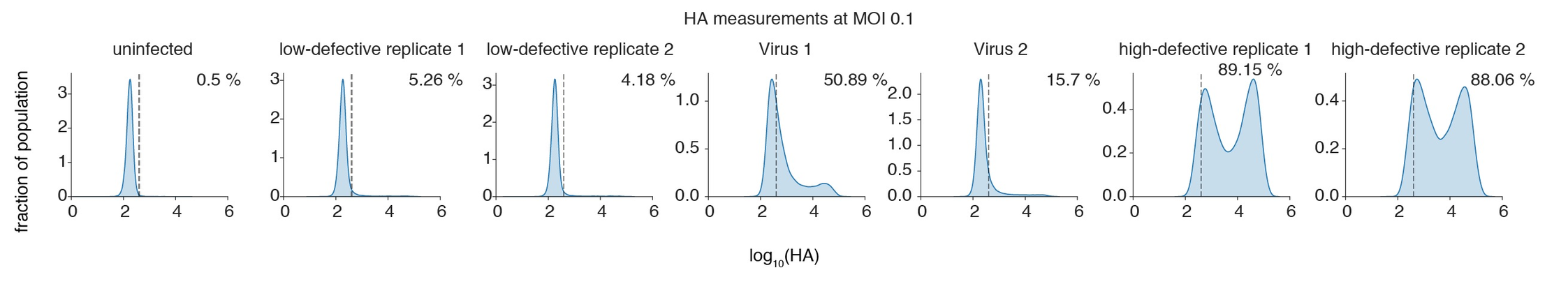

Supplement: S6 Fig — A549 cells were infected with the indicated populations, and, at 9h post-infection, stained with a monoclonal antibody against viral HA protein. Virus 1 and Virus 2 were the two populations used in experiments in Fig 4. Populations were grown as described in Materials and methods. The dotted line indicates the gate at which positivity was called, in this case the 99.5th percentile of the uninfected control. (TIF) [file ppat.1010125.s011.tif]

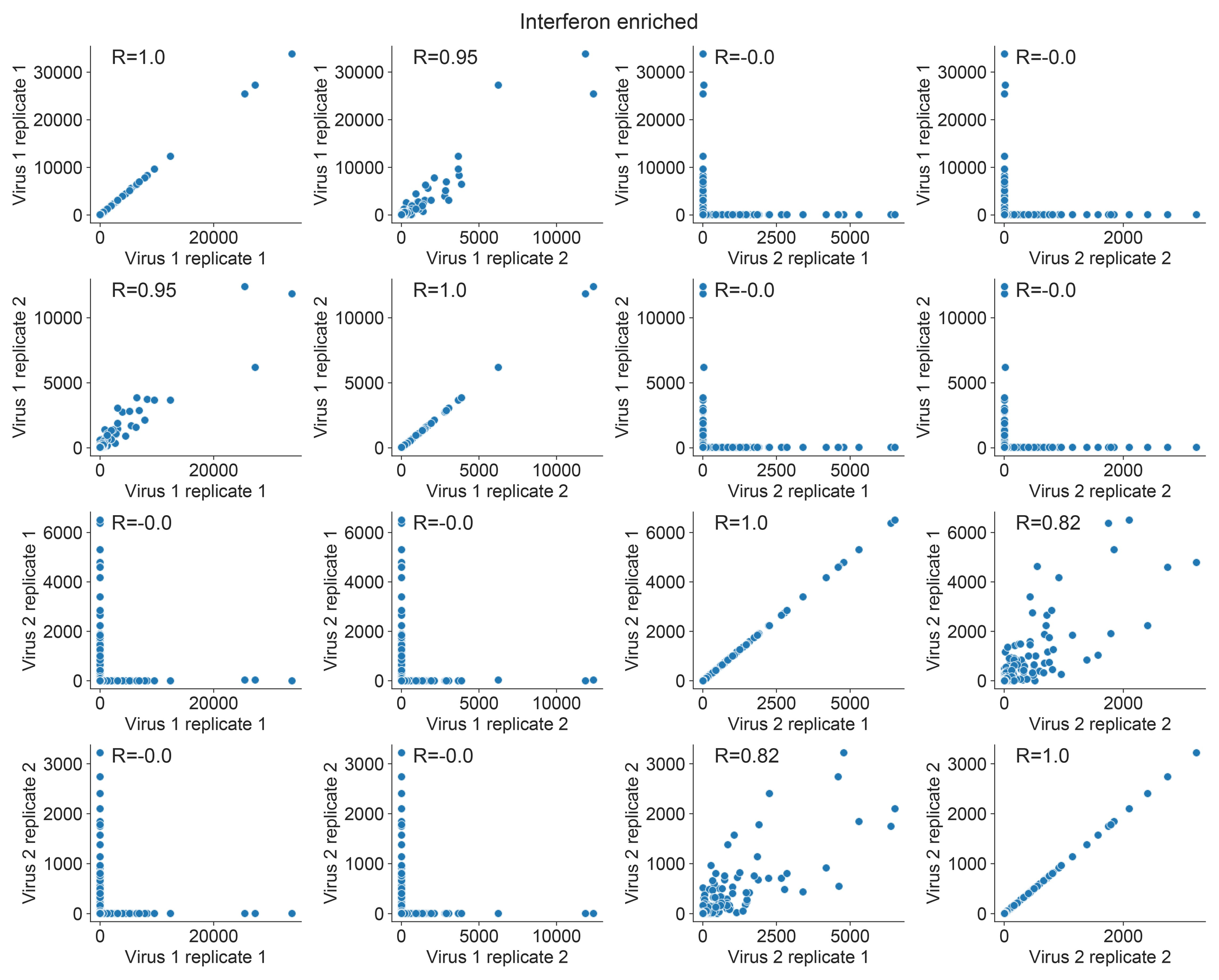

Supplement: S7 Fig — Numbers represent raw counts of deletion-spanning junctions. Each point is a unique deletion. Virus 1 and Virus 2 were the two biologically-independent populations analyzed for Fig 4B. (TIF) [file ppat.1010125.s012.tif]

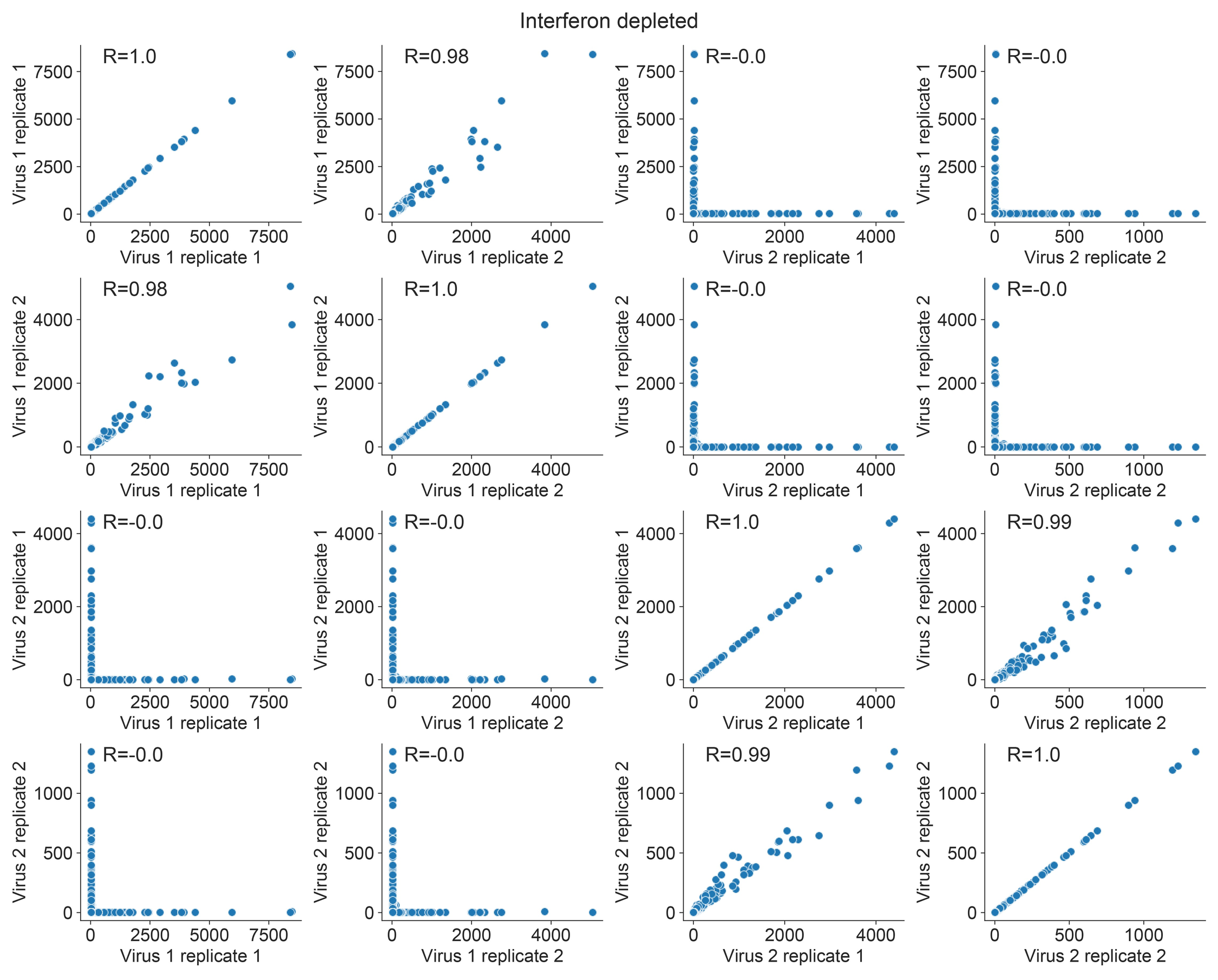

Supplement: S8 Fig — Numbers represent raw counts of deletion-spanning junctions. Each point is a unique deletion. Virus 1 and Virus 2 were the two biologically-independent populations analyzed for Fig 4B. (TIF) [file ppat.1010125.s013.tif]

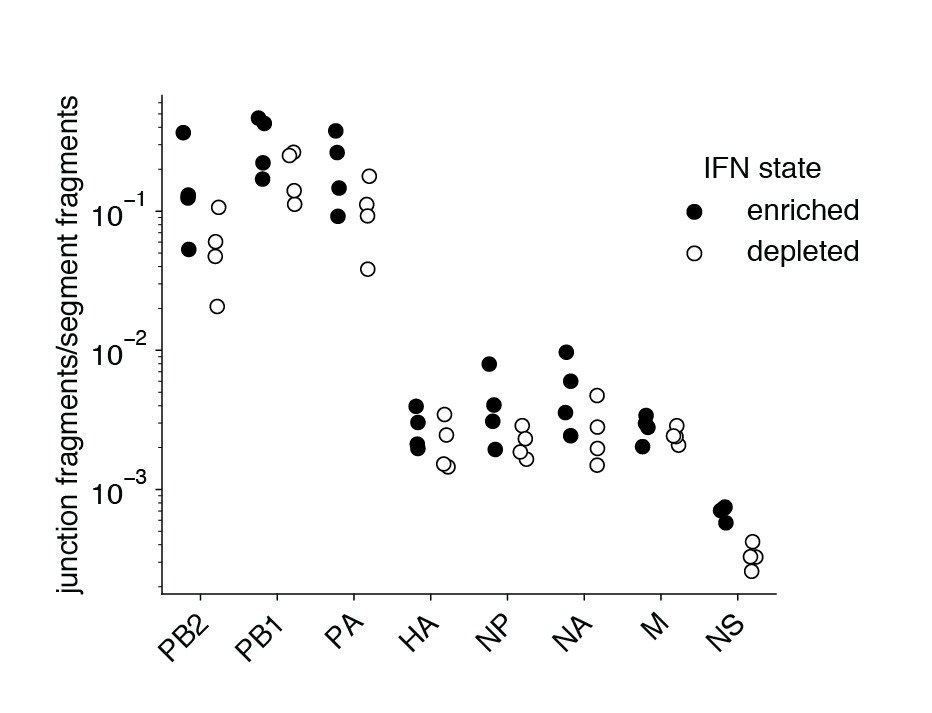

Supplement: S9 Fig — Total number of junction-spanning fragments normalized to the total number of fragments mapped per influenza genomic segment. n = 4. (TIF) [file ppat.1010125.s014.tif]

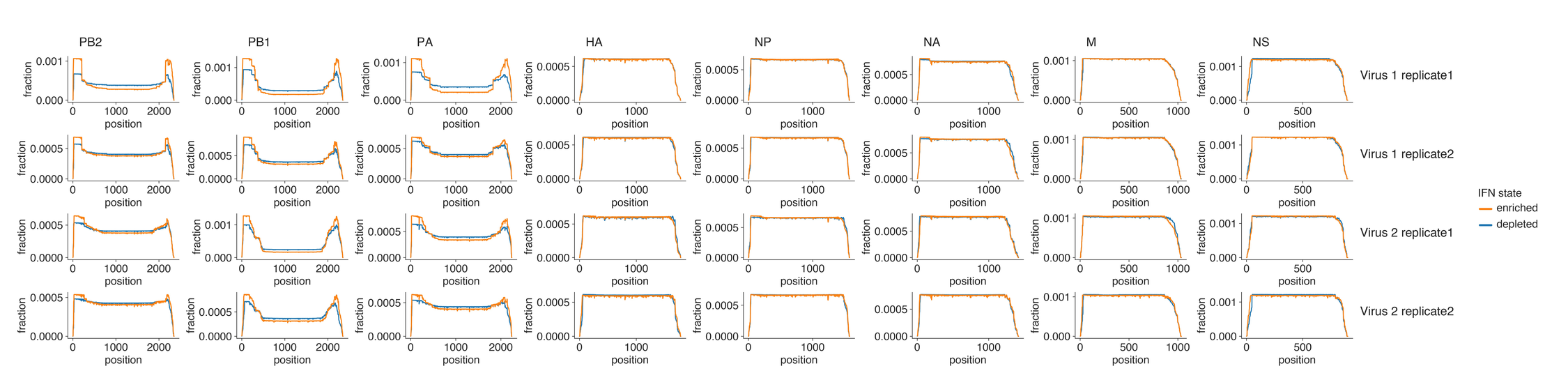

Supplement: S10 Fig — Depth per base corrected to fractional depth, area under each line sums to 1. Increased depth at 5’ and 3’ ends of polymerase segments consistent with increased fractions of deletions. (TIF) [file ppat.1010125.s015.tif]

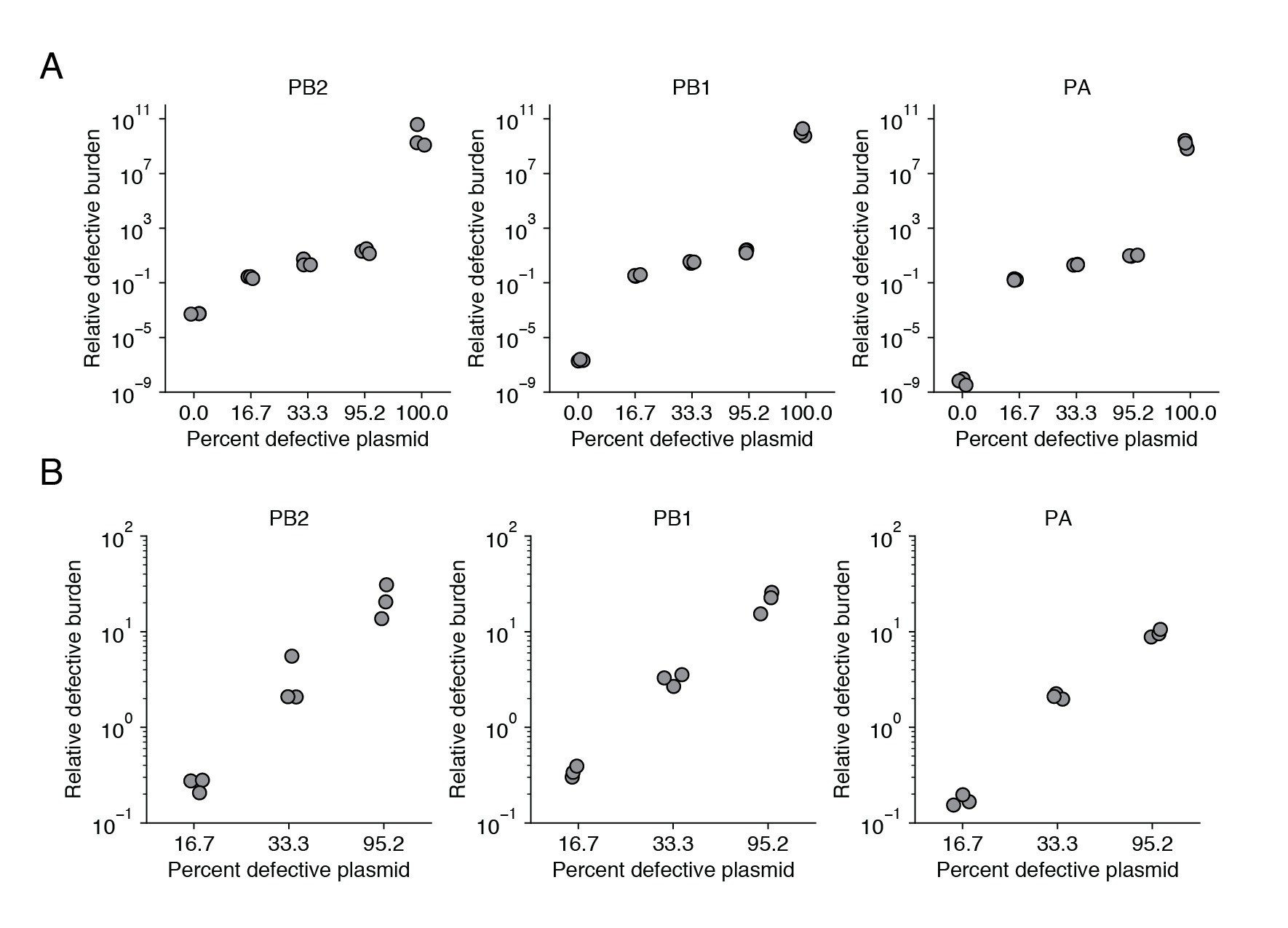

Supplement: S11 Fig — (A)Plasmids containing full-length PB2, PB1, and PA, and those containing PB2316:267, PB1177:385, and PA328:137 were mixed at the indicated molar ratios and 0.02 ng of the resultant mixture was analyzed by qPCR. Values represent the ratio of signal of our defective-spanning qPCR corrected for full-length-only signal. (B) Same as (A) but only intermediate values to show capacity to discriminate between extremes. (TIF) [file ppat.1010125.s016.tif]

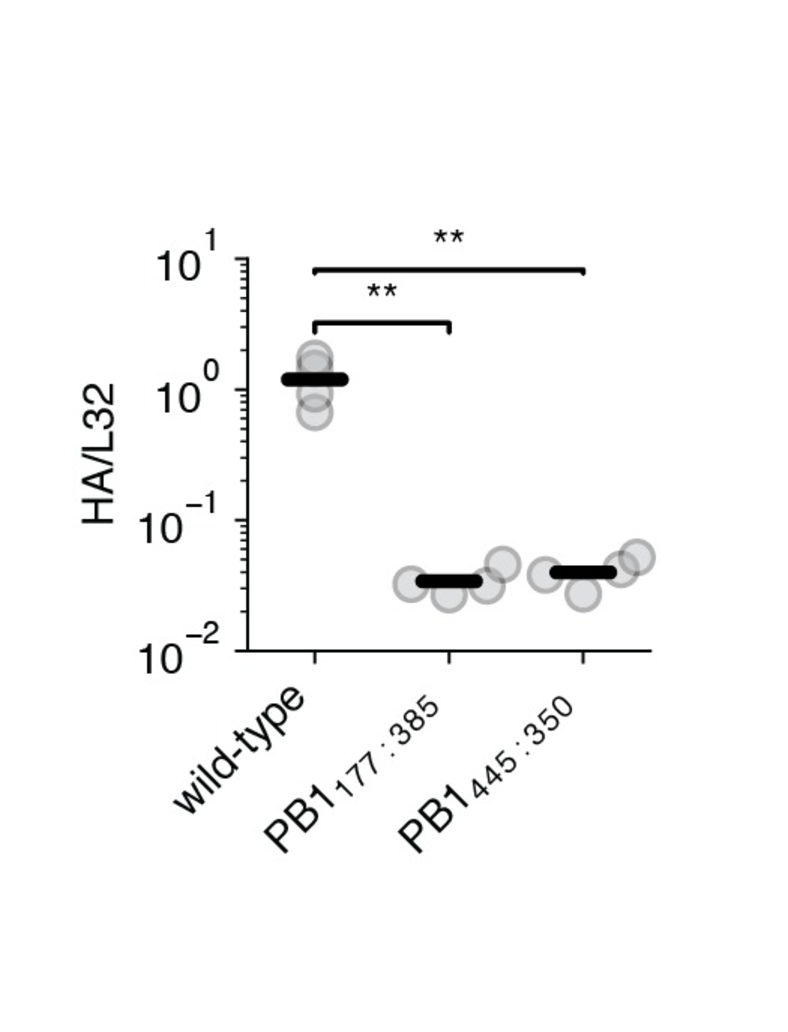

Supplement: S12 Fig — qPCR measuring levels of HA transcript as normalized to the housekeeping control L32 for the indicated influenza mutants infecting A549 cells at an MOI of 0.5 at 8h post-infection. The significant difference between PB1 deletion variants is not due to incorrect dosage, as shown by similar levels of influenza transcripts. Neither PB1 mutant is replication-competent, as shown by the reduced influenza transcript levels compared to a wild-type control. Asterisks represent significantly different values in all pairwise comparisons, two-tailed t-test, using Benjamini-Hochberg multiple-hypothesis correction at an FDR of 0.05. n = 4. (TIF) [file ppat.1010125.s017.tif]

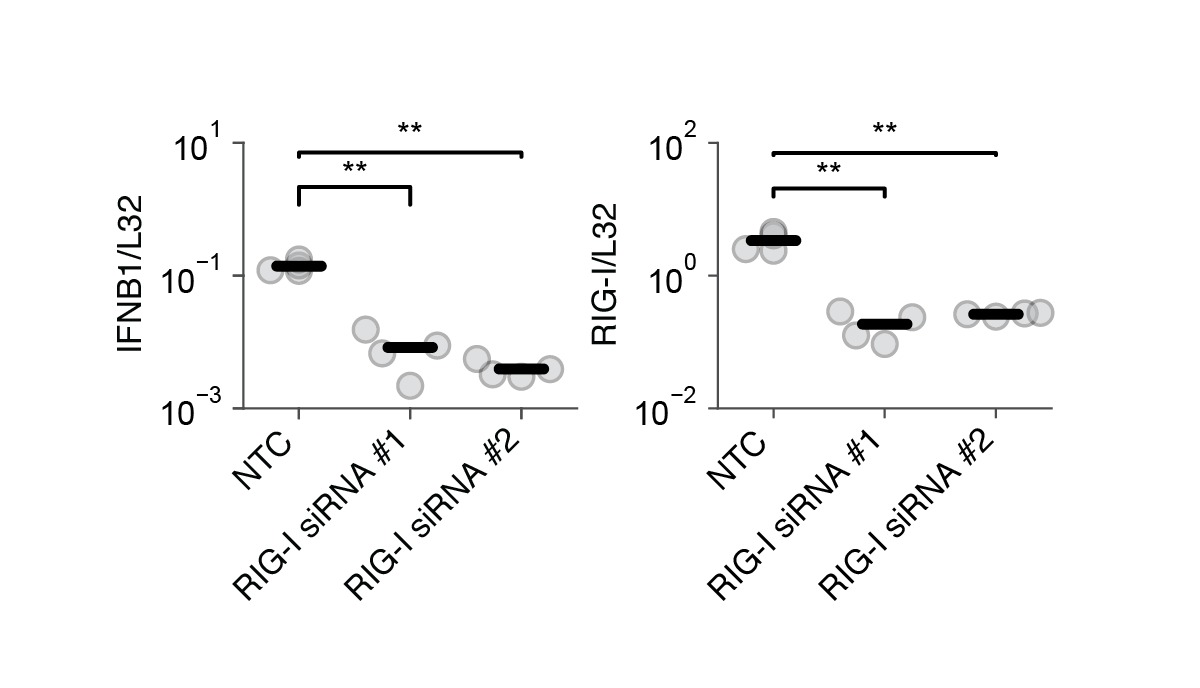

Supplement: S13 Fig — Cells were transfected with the indicated siRNA. 48 hours after transfection, cells were infected with PB1177:385 at an MOI of 0.5, and RNA was harvested at 14 hours and analyzed by qPCR. Asterisks represent significantly different values in all pairwise comparisons, two-tailed t-test, using Benjamini-Hochberg multiple-hypothesis correction at an FDR of 0.05. (TIF) [file ppat.1010125.s018.tif]

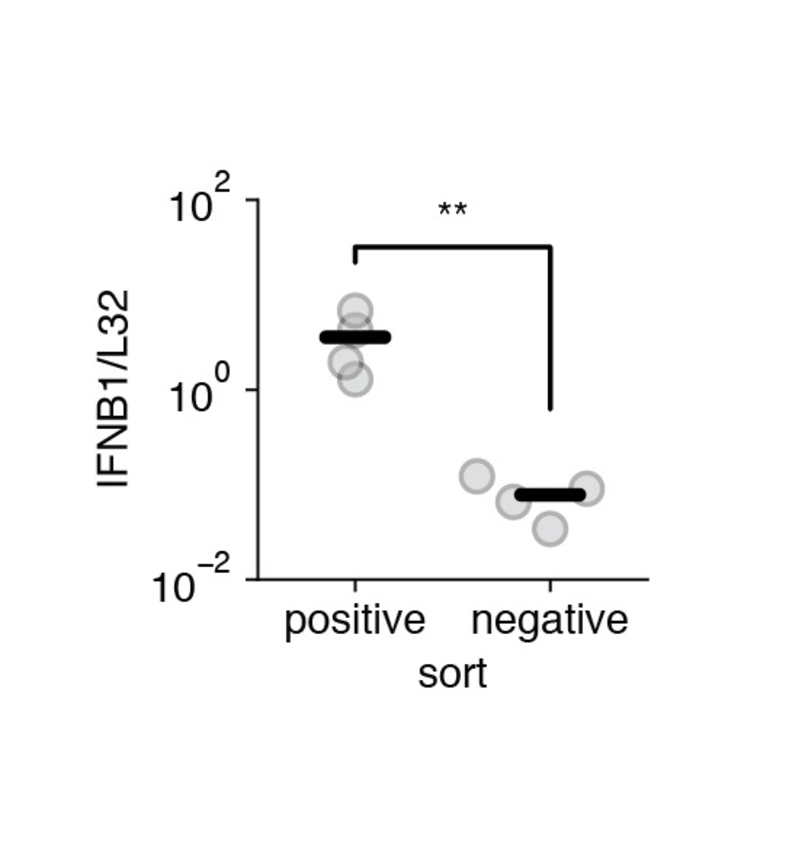

Supplement: S14 Fig — Asterisks indicate significantly increased IFNB1 transcript, two-tailed t-test p < 0.05. n = 3. (TIF) [file ppat.1010125.s019.tif]

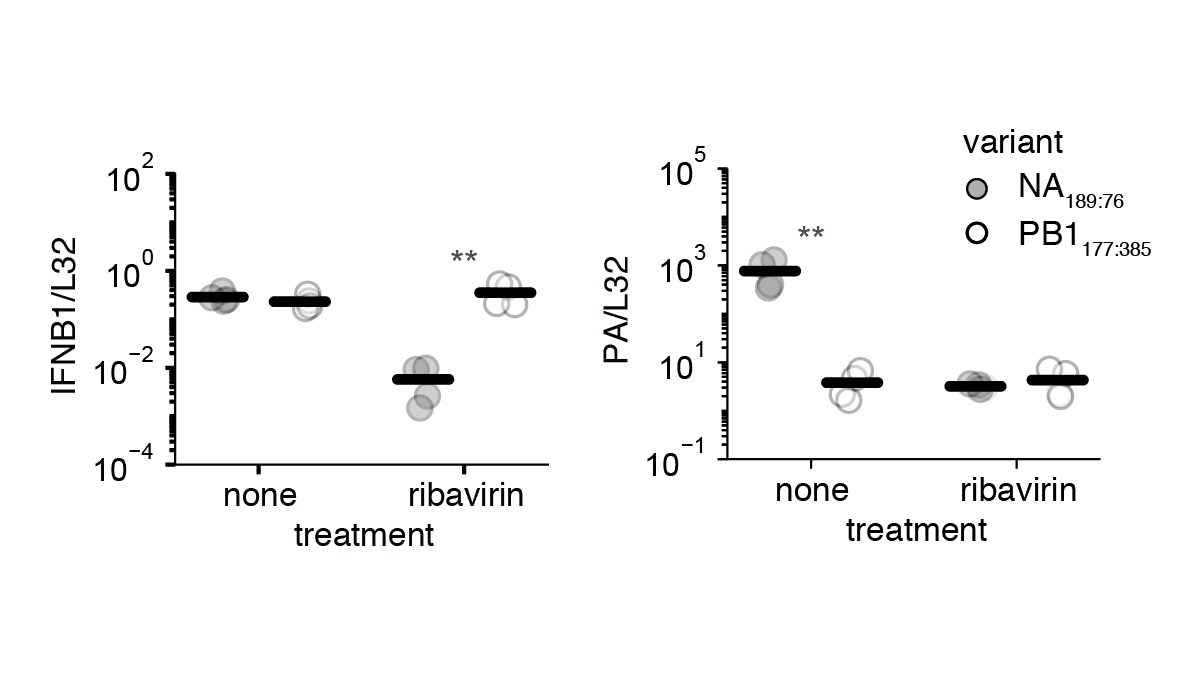

Supplement: S15 Fig — (left) qPCR against IFNB1 as corrected by the housekeeping gene L32 demonstrates that, even with genome replication NA189:76 is no more stimulatory than PB1177:385, and when replication is suppressed, it is actually less stimulatory. (right) qPCR against PA, as corrected by the housekeeping gene L32, was used to validate the suppression of viral replication by the antiviral nucleoside ribavirin, and matched dose between our variants. A549 cells were pretreated with 200 μM ribavirin for 2h and then infected at an MOI of 0.5. RNA was harvested for analysis after 14 hours of infection. Astrisks indicate conditions wherein signal was significantly different between variants. Two-tailed t-test. n = 4. (TIF) [file ppat.1010125.s020.tif]

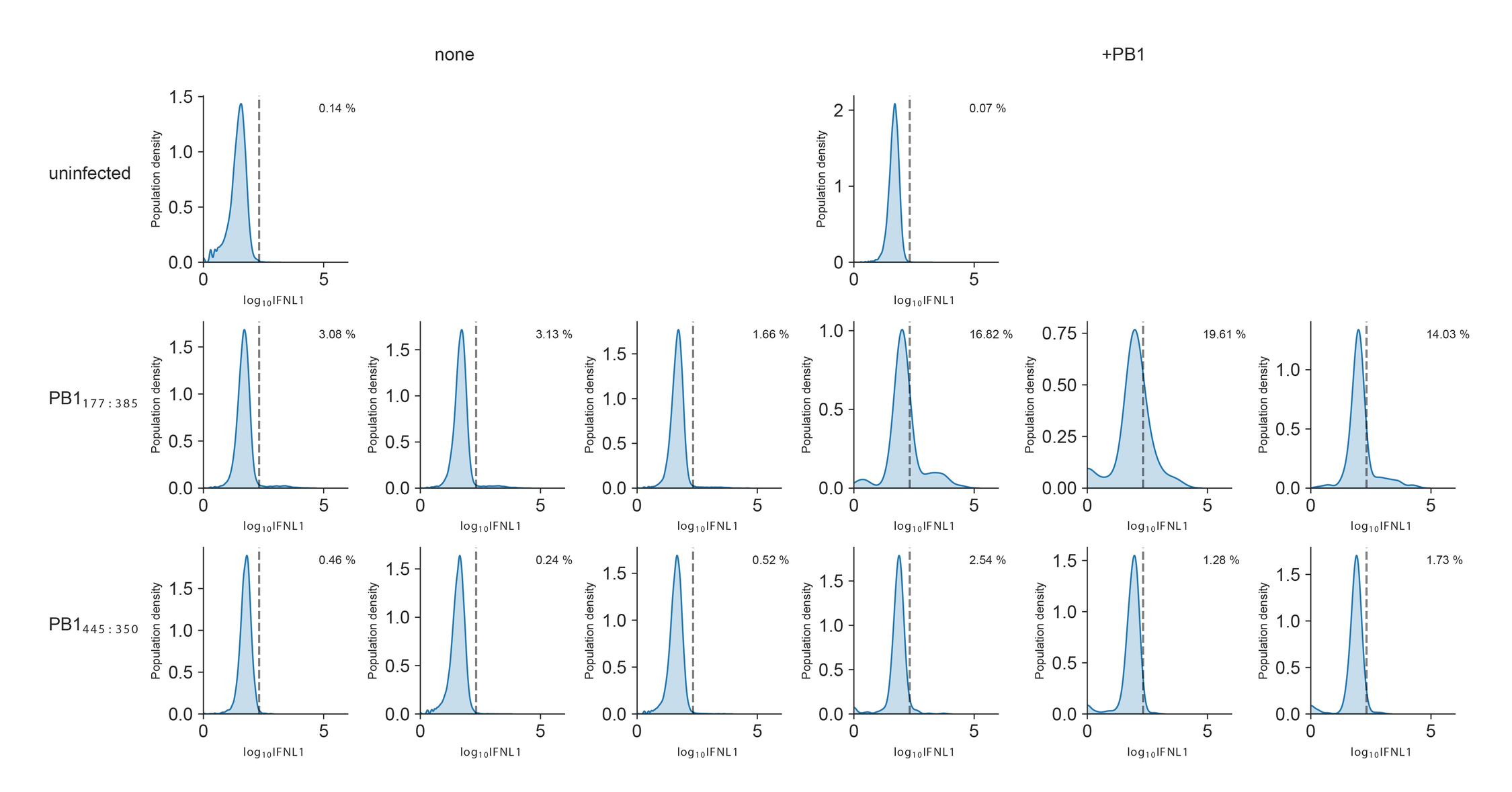

Supplement: S16 Fig — Individual replicates shown. Dotted line represents positivity threshold Gating drawn on the uninfected control before application to infected samples, set at the 99.9th percentile of uninfected. Data shown as kernel density estimates with Gaussian distributions. n = 3. (TIF) [file ppat.1010125.s021.tif]

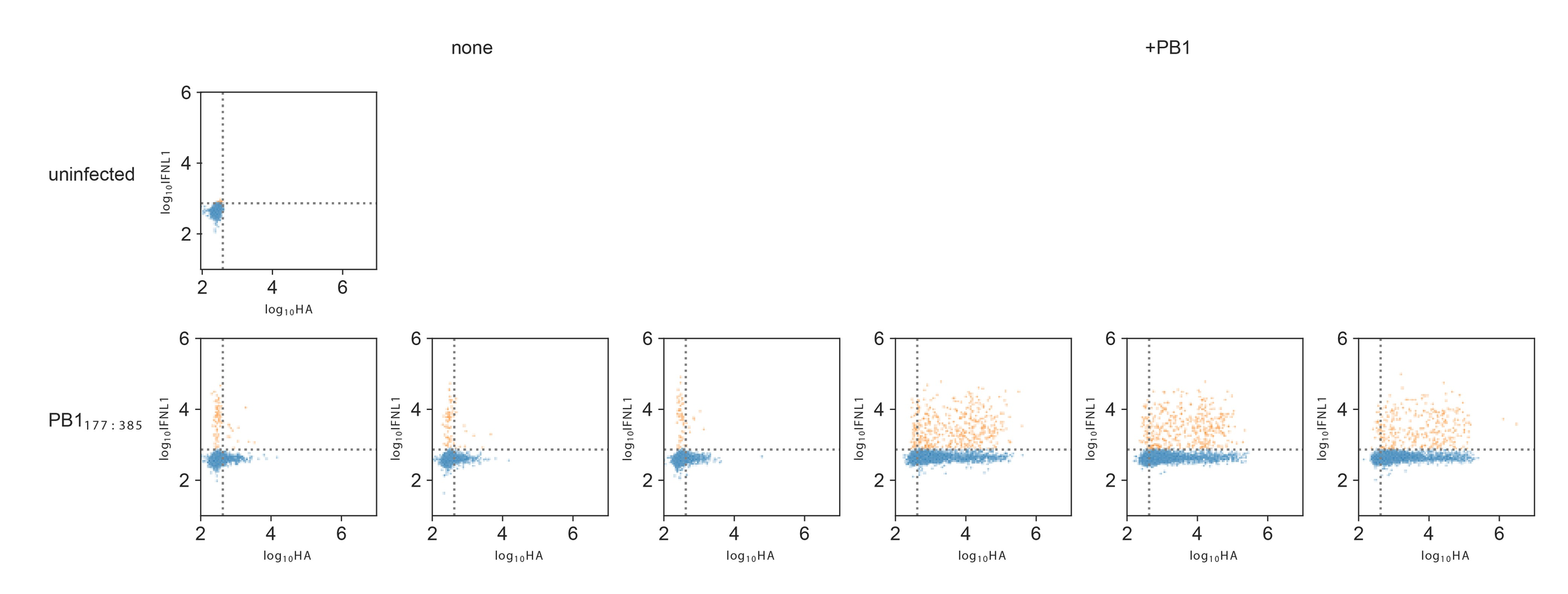

Supplement: S17 Fig — Individual replicates shown. Dotted line represents gating. Gating drawn on the uninfected control before application to infected samples, set at the 99.9th percentile of uninfected. Data were subsampled to 5000 events and shown as individual points. Points in orange were called as interferon-positive. n = 3. (TIF) [file ppat.1010125.s022.tif]

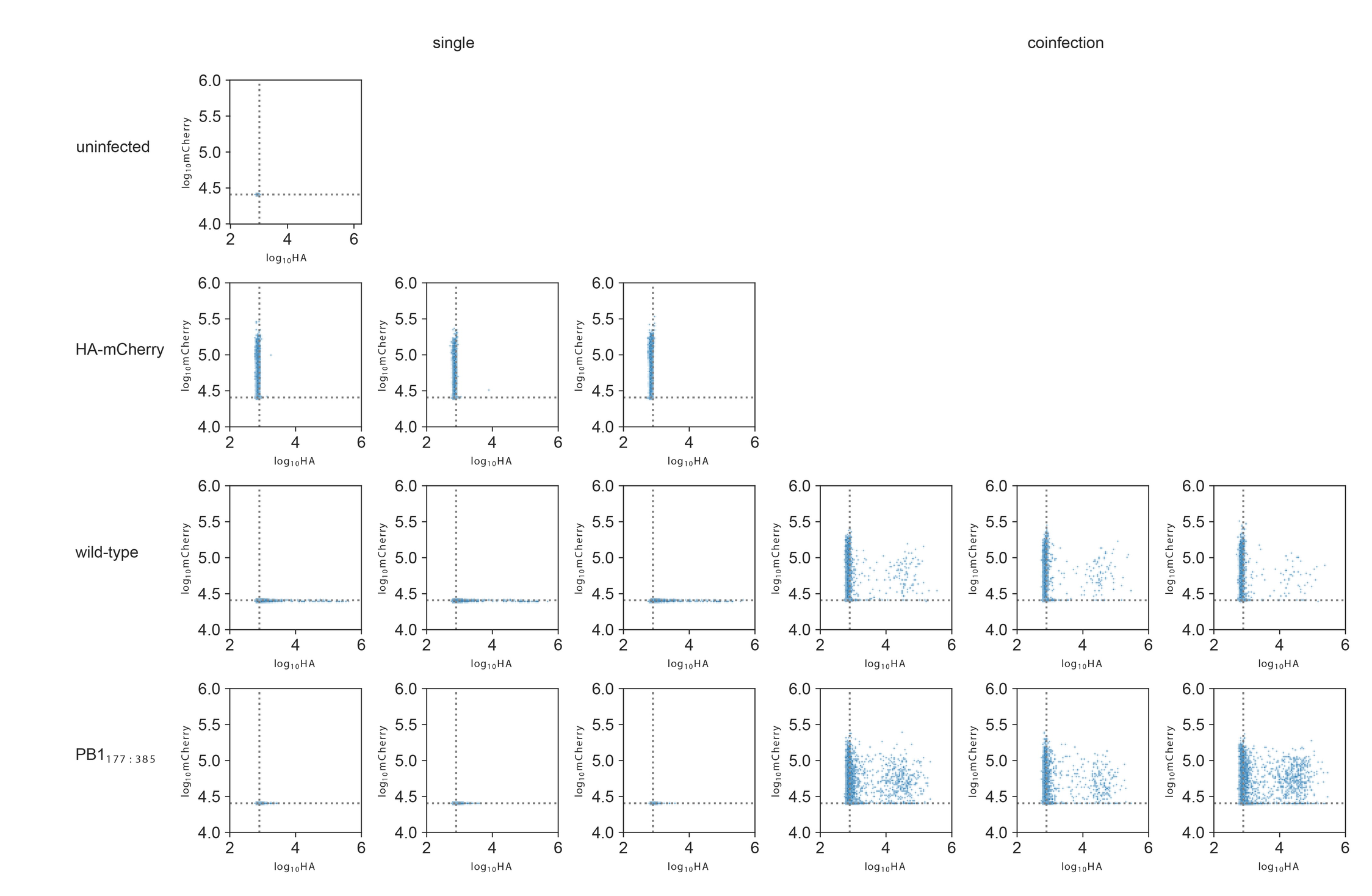

Supplement: S18 Fig — Individual replicates shown. Dotted line represents gating. Gating drawn on the uninfected control before application to infected samples, set at the 99.95th percentile of uninfected. Data were subsampled to 5000 events and shown as individual points. n = 3. (TIF) [file ppat.1010125.s023.tif]

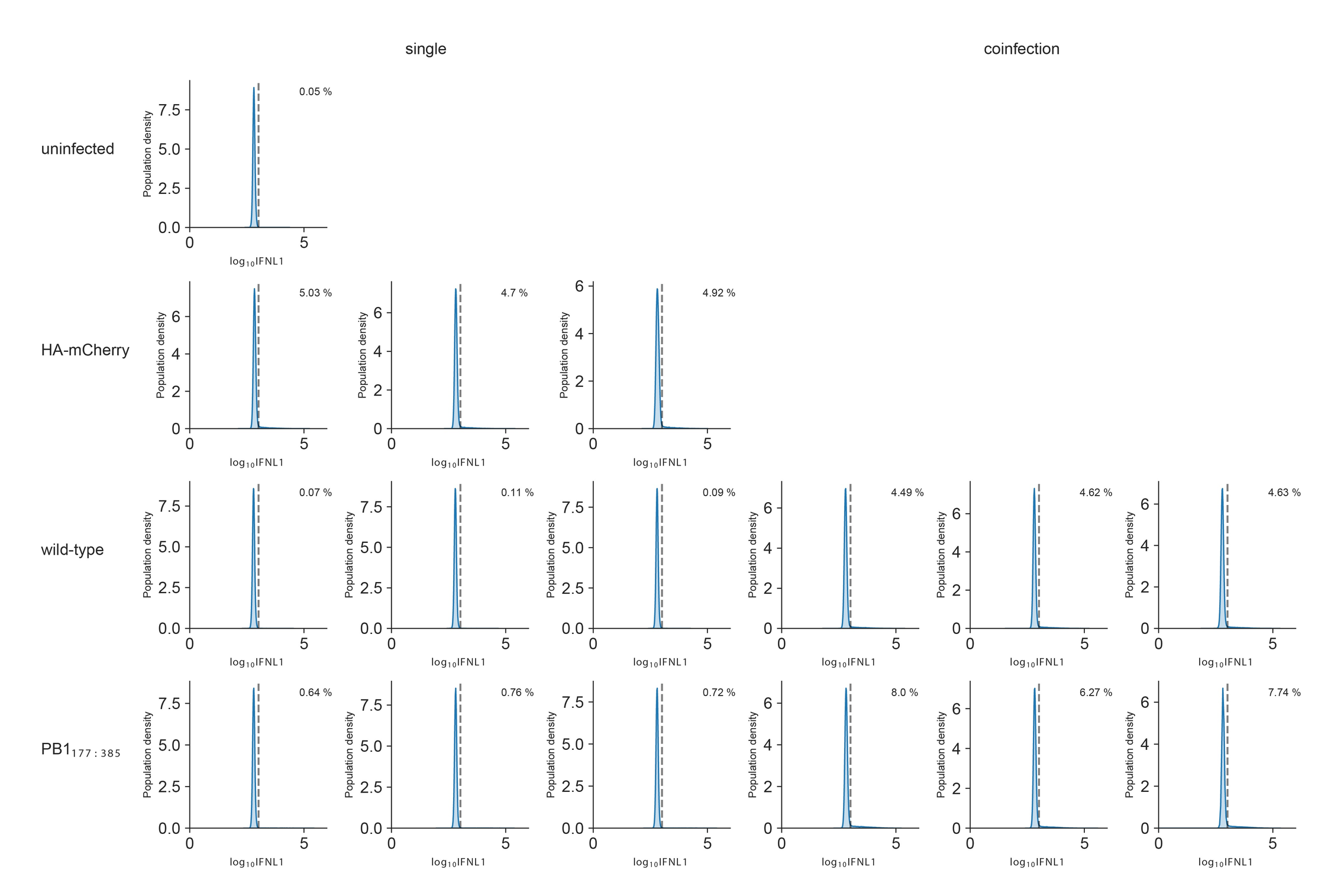

Supplement: S19 Fig — Individual replicates shown. Dotted line represents gating. Gating drawn on the uninfected control before application to infected samples, set at the 99.95th percentile of uninfected. Data shown as kernel density estimates with Gaussian distributions. n = 3. (TIF) [file ppat.1010125.s024.tif]

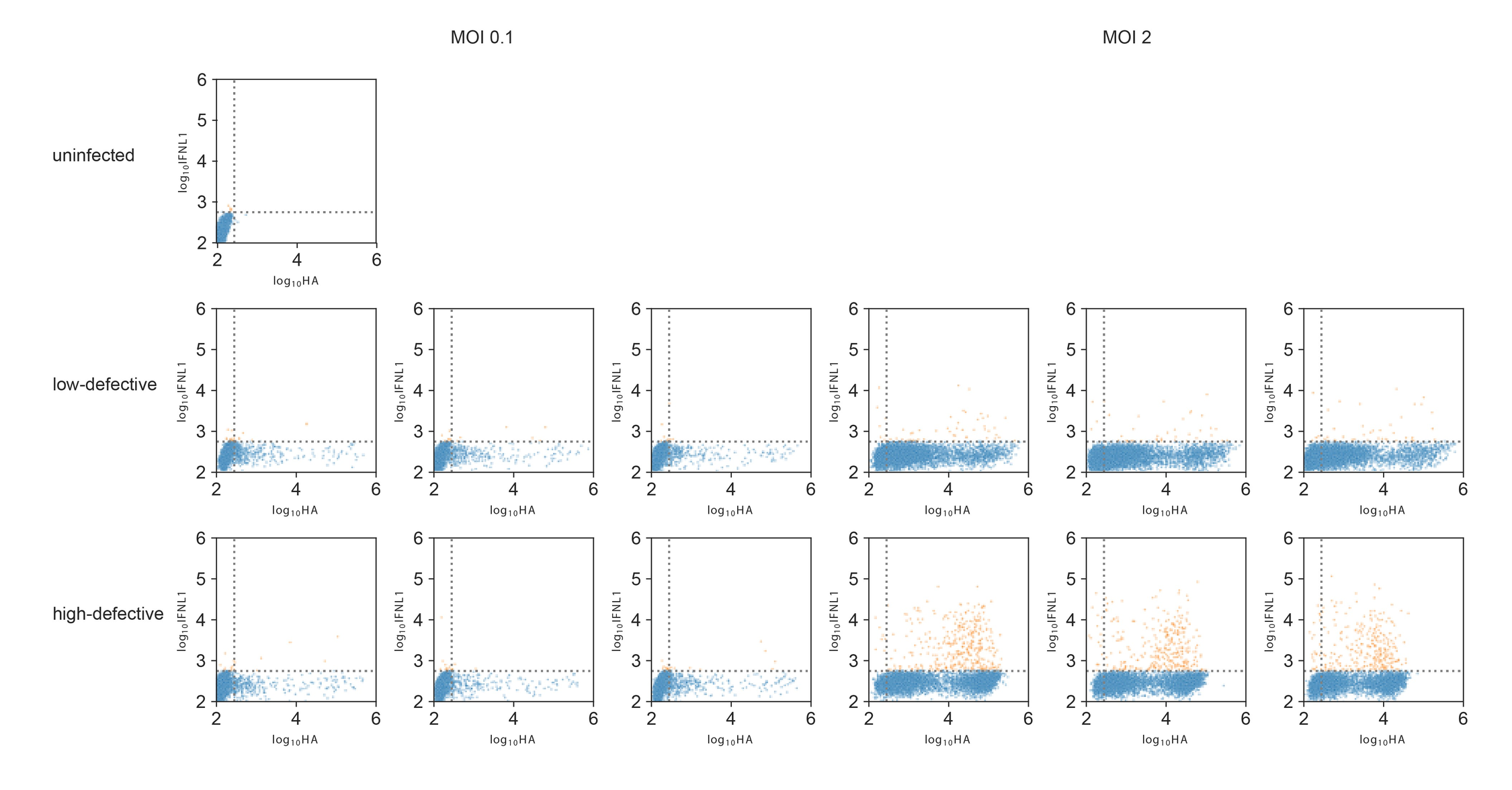

Supplement: S20 Fig — Individual replicates shown. Dotted line represents gating. Gating drawn on the uninfected control before application to infected samples, set at the 99.9th percentile of uninfected. Data were subsampled to 5000 events and shown as individual points. Points in orange were called as interferon-positive. n = 3. (TIF) [file ppat.1010125.s025.tif]
